# A systematic review of machine learning models for predicting outcomes of stroke with structured data

**DOI:** 10.1371/journal.pone.0234722

**Published:** 2020-06-12

**Authors:** Wenjuan Wang, Martin Kiik, Niels Peek, Vasa Curcin, Iain J. Marshall, Anthony G. Rudd, Yanzhong Wang, Abdel Douiri, Charles D. Wolfe, Benjamin Bray

**Affiliations:** 1 School of Population Health & Environmental Sciences, Faculty of Life Science and Medicine, King’s College London, London, United Kingdom; 2 School of Medical Education, Faculty of Life Science and Medicine, King’s College London, London, United Kingdom; 3 Division of Informatics, Imaging and Data Science, School of Health Sciences, University of Manchester, Manchester, United Kingdom; 4 NIHR Manchester Biomedical Research Centre, Manchester Academic Health Science Centre, University of Manchester, Manchester, United Kingdom; 5 NIHR Biomedical Research Centre, Guy's and St Thomas' NHS Foundation Trust and King's College London, London, United Kingdom; 6 NIHR Applied Research Collaboration (ARC) South London, London, United Kingdom; Karolinska Institutet, SWEDEN

## Abstract

**Background and purpose:**

Machine learning (ML) has attracted much attention with the hope that it could make use of large, routinely collected datasets and deliver accurate personalised prognosis. The aim of this systematic review is to identify and critically appraise the reporting and developing of ML models for predicting outcomes after stroke.

**Methods:**

We searched PubMed and Web of Science from 1990 to March 2019, using previously published search filters for stroke, ML, and prediction models. We focused on structured clinical data, excluding image and text analysis. This review was registered with PROSPERO (CRD42019127154).

**Results:**

Eighteen studies were eligible for inclusion. Most studies reported less than half of the terms in the reporting quality checklist. The most frequently predicted stroke outcomes were mortality (7 studies) and functional outcome (5 studies). The most commonly used ML methods were random forests (9 studies), support vector machines (8 studies), decision trees (6 studies), and neural networks (6 studies). The median sample size was 475 (range 70–3184), with a median of 22 predictors (range 4–152) considered. All studies evaluated discrimination with thirteen using area under the ROC curve whilst calibration was assessed in three. Two studies performed external validation. None described the final model sufficiently well to reproduce it.

**Conclusions:**

The use of ML for predicting stroke outcomes is increasing. However, few met basic reporting standards for clinical prediction tools and none made their models available in a way which could be used or evaluated. Major improvements in ML study conduct and reporting are needed before it can meaningfully be considered for practice.

## Introduction

Stroke is the second leading cause of mortality and disability adjusted life years in the world [1,2]. Both the outcomes and presentation of stroke can be extremely varied and timely assessment is essential for optimal management. The complexity of a condition such as stroke potentially lends itself well to the use of ML methods which are able to incorporate a large variety of variables and observations into one predictive framework without the need for preprogrammed rules. There has been increasing interest in the use of ML to predict stroke outcomes, with the hope that such methods could make use of large, routinely collected datasets and deliver accurate personalised prognoses.

While papers applying ML methods to stroke are published regularly, the main focus of these has been on stroke imaging application [3–5]. As far as we are aware, there have been no reviews of studies which have developed ML models to predict stroke outcomes from structured data specifically. The goal of the review was to identify gaps in the literature, critically appraise the reporting and methods of the algorithms and provide the foundation for a wider research program focused on developing novel machine learning based predictive algorithms in stroke care.

## Methods

This is a systematic review which was registered with the international prospective register of systematic reviews (PROSPERO) (CRD42019127154): a database of systematic review protocols, maintained by the Centre for Reviews and Dissemination at the University of York. The PRISMA [6] statement was followed as a reporting guideline. Risk of bias and quality of the studies were not assessed because the objective of this paper was to be descriptive, not to draw conclusions about the validity of estimates of predictive accuracy from the included studies. The reporting quality was assessed according to TRIPOD [7] with a few terms adjusted to fit ML methods (See Table 1 for explanations of ML terms).

**Table 1:** Notations of special machine learning terms.

Term	Explanation
Supervised learning	A subgroup of ML models that requires both predictors and outcomes (labels)
Unsupervised learning	A subgroup of ML models meant to find previously unknown patterns in data without pre-existing labels
Feature	Predictor or variable in a ML model
Feature selection	Variable selection or attribute selection
Generalisation ability	The ability of a model to generalise the learned pattern to new data
Over-fitting	A model corresponds too closely or exactly to a particular set of data, and may fail to fit new data
Missing data mechanism	Three missing-data mechanisms: missing completely at random (MCAR), missing at random (MAR), and missing not at random (MNAR)
Imputation	The process of replacing missing data with substituted values
Training	The learning process of the data pattern by a model
Testing	A validation set used for testing the model
LASSO	Least Absolute Shrinkage and Selection Operator: a regression technique that performs both variable selection and regularization
Support Vector Machine (SVM)	A supervised classifier that seeks to find the best hyperplane to separate the data
Naïve Bayes (NB)	A family of simple "probabilistic classifiers" based on applying Bayes' theorem with strong (naïve) independence assumptions between the features
Bayesian Network (BN)	A type of probabilistic graphical model that uses Bayesian inference for probability computations
k-nearest neighbours (kNN)	A type of instance-based learning, where the predictionis only approximated locally with the k nearest neighbours
Artificial Neural Network (ANN)	A computational model based on a collection of connected units or nodes called artificial neurons, which loosely model the neurons in a biological brain
Decision Tree	A tree with a set of hierarchical decisions which eventually gives a final decision
Random Forest (RF)	An ensemble learning method that uses a multitude of decision trees
Super learner	A stacking algorithm using cross-validated predictions of other models and assigning weights to these predictions to optimise the final prediction
Adaptive network based fuzzy inference system (ANFIS)	A fuzzy Sugeno model put in the framework of adaptive systems to facilitate learning and adaptation
Xgboost	A decision-tree-based ensemble ML algorithm that uses a gradient boosting framework
Adaptive Boosting, (Adaboost)	An algorithm used in combination with others to convert a set of weak classifiers into a strong one
Parameters	Coefficients of a model that need to be learned from the data
Hyperparameters	Configurations of a model which are often selected and set before training the model
Validation	The process of a trained model evaluated with a testing dataset
Discrimination	The ability of a model to separate individual observations in multiple classes
Calibration	Adjusting the predicted probability from the model to more closely match the observed probability in the test set
Cross-validation (CV)	A model validation technique for assessing how the results of a statistical analysis (model) will generalize to an independent data set
Leave One Out CV	A performance measurement approach that uses one observation as the validation set and the remaining observations as the training set
Leave One Centre Out CV	A performance measurement approach that uses observations from one centre as the validation set and the remaining observations as the training set
Bootstrapping	Resampling multiple new datasets with replacement from the original data set

### Search strategy

We searched PubMed and Web of Science for studies on prediction models for stroke outcomes using ML, published in English between 1990 and March 2019. We combined published PubMed search filters for stroke [8], ML [9], and prediction models [10]. To ensure consistency in the searches in both databases, these PubMed filters were translated to Web of Science together with the support of a librarian. We verified the search strategy (S1 Text) with a validation set of seven publications identified manually by the researchers across PubMed and Web of Science and the results of our database queries included all the seven papers in this validation set.

### Study selection

We assessed the eligibility of the studies returned by the searches through a two-stage screening process. We first screened the titles and abstracts of all articles. Two authors (WW and MK) independently screened 50% of articles each and a random sample of 10% in duplicate. Any disagreement was solved through discussion, involving a third author (BB) if necessary. For all studies deemed relevant, the full text was reviewed using the same screening procedure as in the first stage.

Studies were eligible if they adhered to the following inclusion criteria:

- Focusing on predicting clinical outcomes of stroke, excluding studies predicting the occurrence of stroke
- Using structured patient level health data (electronic health records, insurance claims data, registries, cohort studies data, or clinical trials data), excluding studies using text or imaging data
- Primary research only, excluding reviews
- Complete paper available rather than just an abstract or notes

### Reporting quality assessment

Reporting guidelines for ML as prediction models are currently not available. TRIPOD was followed as a reporting standard which was originally developed for regression modelling. As mentioned in TRIPOD’s documentation, most terms apply equally to ML methods developed, validated, or updated as prediction tools. We adopted most terms for reporting of methods and results in TRIPOD with two terms adjusted specifically for ML (S1 Table). Reporting of hyperparameter selection if needed was added to 10b (Specify type of model, all model building procedures) and 15a (Present the full prediction model to allow predictions for individuals) was adjusted for the specification of ML models (links to the final model online, coding of predictors, code, final parameters/coefficients, and with the architecture described in full in the article).

### Data extraction

An structured data collection form was developed to aid extraction of items related to: general study characteristics (authors, publication year, type, venue, country under study population, study objective); study population (source of data, single or multi-centre, sample size, features, feature size); data pre-processing methods (handling missing data and unbalanced outcomes, other data pre-processing steps); clinical outcomes; analytical methods (statistical models, ML models, feature selection methods, validation methods, performance measurements); results (feature importance, best performing model) (S2 Table).

Data for all papers were extracted by two authors (WW and MK), with discrepancies resolved by consensus through discussion between and with another author (BB) if necessary.

## Results

**Fig 1:**
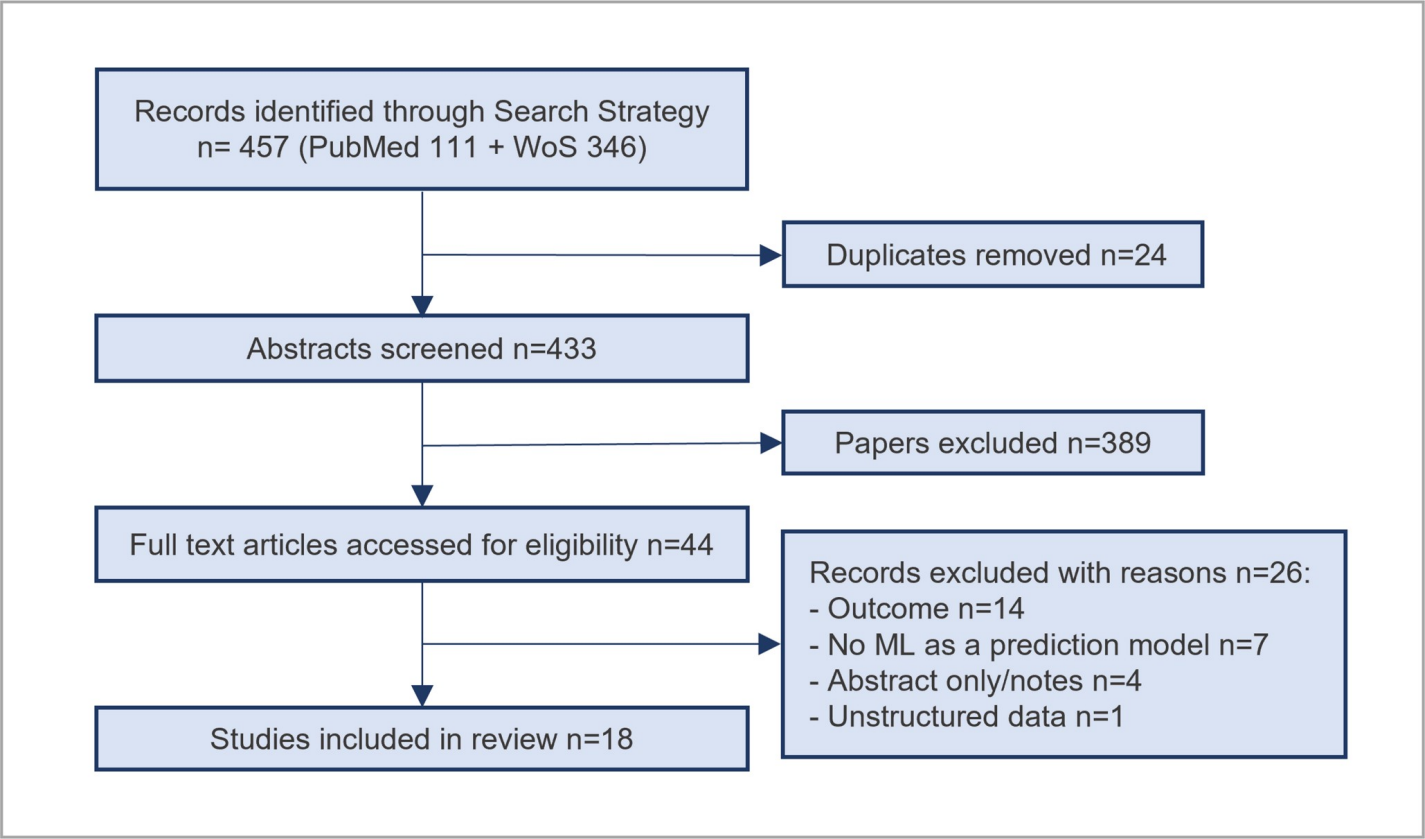
PRISMA flowchart.

We identified 111 studies from PubMed and 346 studies from Web of Science. After the removal of duplicates, as well as abstract and title screening, 44 studies were considered potentially relevant. After full article screening, 18 studies were identified for information extraction (Fig 1).

**Fig 2:**
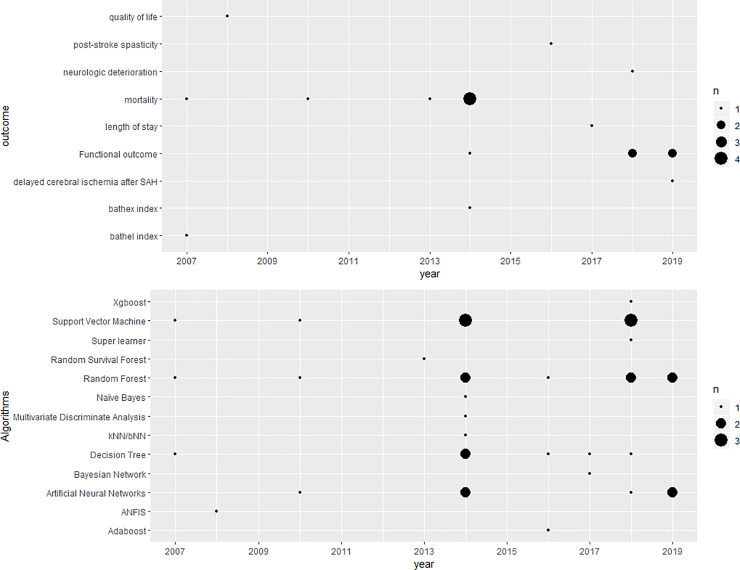
Number of papers published according to the algorithms used (top) and outcomes (bottom) predicted at each year.

Almost all studies (17) were published as peer reviewed publications in biostatistical or clinical journals. All included studies were published after 2007, with almost half (8) published after 2016, and 3 studies were published in 2018 [11–13] and 2019 [14–16] each (Fig 2). In terms of regions under study, UK (3) [17–19], Germany (2) [20,21], Turkey (2) [22,23] and China (2) [13,14] make up half of the sample. Saudi Arabia [24], Australia [25], Korea [15], USA [26], Denmark [27], Netherlands [11], Portugal [12], Taiwan [28] and Japan [16] had one study each. Single centre studies (10) were slightly more common than multi-centre studies (8). For sources of data, half of the studies (9) used registry data while the rest used EHR (4) [13,22,24,28], cohort (3) [14,15,27], and clinical trial data (2) [11,18]. All the included studies focused on developing new models using ML whilst no study validated existing ML based predictive models on independent data. Most of the studies used only variables collected at admission (Table 2) though three studies [11,12,16] explored model performance with information available at different time points.

**Table 2:** A brief summary of the included studies.

Reference	Sample (Feature) size	Outcomes	Predictors/variables/features	Missing values handled	Hyperparameter selection	Validation	Calibration	Best Algorithm	Compared algorithms
Al Taleb et al. 2017	358 (15)	Length of Stay	At admission	Single imputation	Not reported	10-fold CV	No	Bayesian Network	DT (C4.5)
Asadi et al. 2014	107 (8)	90-day binary and 7 scale mRS	At admission	Not reported	No	Training, test, validation for ANN, Nested CV for SVM	No	SVM	ANN, Linear Regression
Liang et al. 2019	435 (4)	90-day binary mRS	Admission, laboratory data	Not reported	Not reported	Training and test split	No	ANN	LR
Heo et al. 2019	2604 (38)	90-day binary mRS	Admission	Complete case analysis	No	Training and test split	No	DNN	RF, LR
Konig et al. 2007	3184 (43)	100-day Bathel Index	first 72h after admission	Complete case analysis	Yes, Grid search	Temporal and external validation	Yes for LR	-	RF, SVM, LR
Celik et al. 2014	570 (22)	10-day mortality	At admission	-	Yes, grid search	5-fold CV	No	LR	ANN
Ho et al. 2014	190 (26)	Discharge mortality	Admission and interventions	Complete case analysis	Not reported	10-fold CV	No	SVM	Naïve Bayes, DT, RF, PCA+SVM, LR
Cox et al. 2016	2580 (72)	Post stroke spasticity	Not clear	Not reported	Not reported	Training, test and validation split	No	RF	DT (CART), Adaboost
Kruppa et al. 2014	3184 (43)	100-day Bathel Index	First 72h after admission data	Complete case analysis	Yes, For KNN, bNN and RF	Temporal and external validation	Yes, Brier score	SVM and LR	K-NN, b-NN, RF
Easton et al. 2014	933 (-)	Short/very short mortality	Not clear	Not reported	Yes, DT is pruned	Training and test split	No	-	Naïve Bayes, DT, LR
Mogensen and Gerds 2013	516 (12)	3-month/1-year/3-year/5-year mortality	Admissiondata	Complete case analysis	No, manually set up	Bootstrap CV	Yes, Brier score	-	Pseudo RF, Cox Regression, and Random survival forest
Van Os et al. 2018	1383 (83)	Good reperfusion socre, 3-month binary mRS	Admission, laboratory and treatment data	Multiple imputation by chained equations	Yes, nested CV with random grid search	Nested CV	No	-	RF, SVM, ANN, super learner, LR
Peng et al. 2010	423 (10)	30-day mortality	Admission, laboratory, radiographic data	No missing values	Yes, empirically	4-fold CV	No	RF	ANN, SVM, LR
Tokmakci et al. 2008	70 (6)	Quality of life	Admissiondata	Not reported	Not reported	Training and test split	No		ANFIS
Monteiro et al. 2018	425 (152)	3-month binary mRS	Admission/2 hours/24 hours/7 days data	single imputation	Yes, Grid search	10-fold CV	No	RF and Xgboost	DT, SVM, RF, LR (LASSO)
Tjortjis et al. 2007	671 (37)	2-month mortality	Admission data	Cases discarded with missing outcomes	Yes, pruned	Training and test split	No	DT (T3)	DT (C4.5)
Lin et al. 2018	382 (5)	Neurologic deterioration	Admission and laboratory data	Not reported	Yes, CV	Training and test split	No	-	SVM
Tanioka et al. 2019	95 (20)	Delayed cerebral ischemic after SAH	Admission/1-3 days variables	Complete case analysis	Yes, Grid search	Leave one out CV	No	-	RF

**Fig 3:**
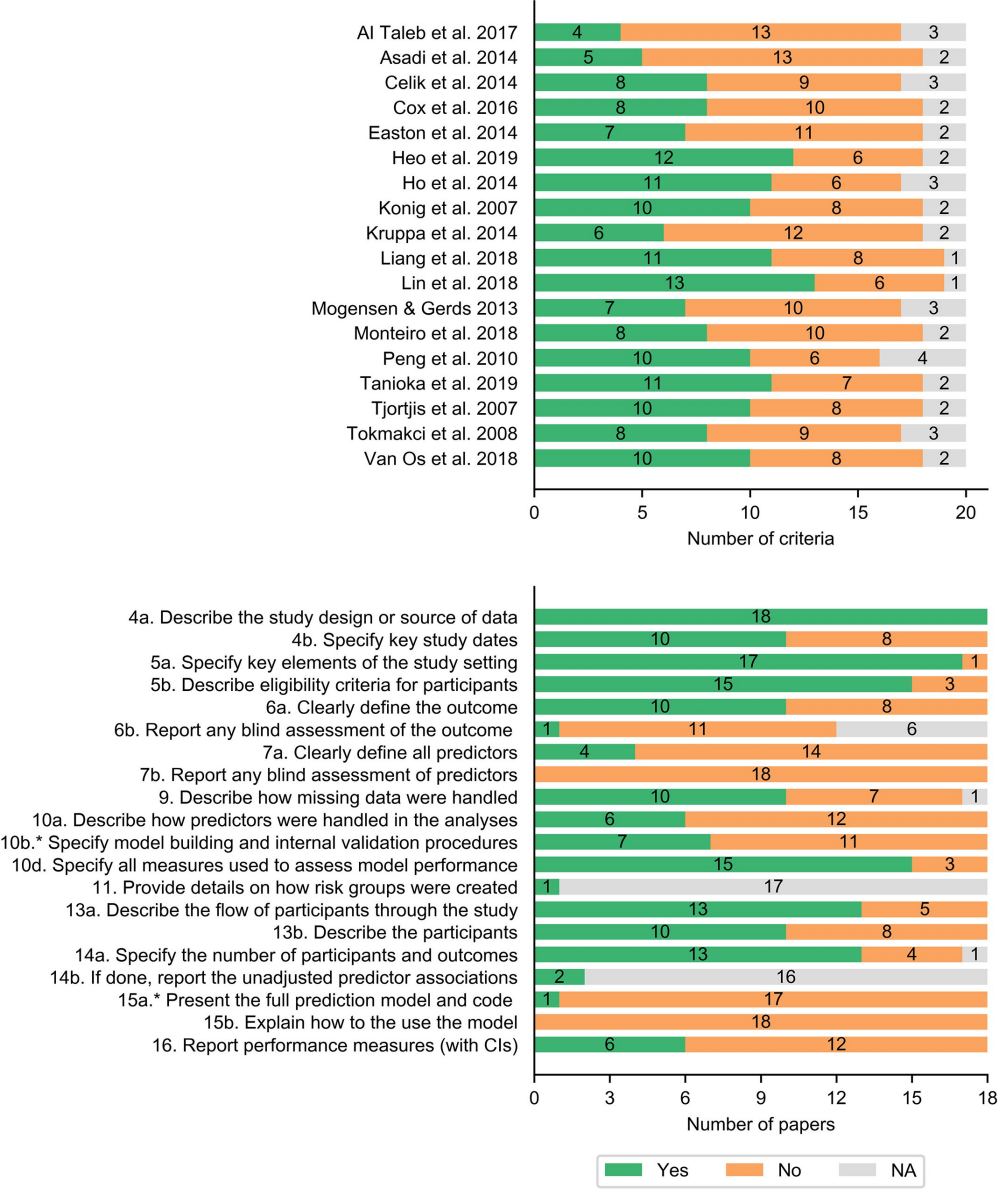
**Number of terms reported in each study (top) and number of studies reported for each assessment term (bottom).** * indicates criteria adjusted for ML models.

Twenty terms were assessed for each study, including thirteen terms for methods and seven terms for results. Half of the studies reported less than half of the terms in the checklist (excluding NA) and the other half of the studies reported less than around two thirds of the terms (Fig 3). The study design and source of data (4a), study setting (5a), eligibility criteria for participants (5b), measures to assess the model (10d), flow of participants (13a), number of participants and outcomes (14a) were relatively better reported (with more than 13 studies) (Fig 3). Blind assessment of outcome and predictors (6b and 7b), presentation of the full model (15a), and explanation on how to use the model (15b) were not reported in almost any of the studies. Definition of all predictors (7a) and description of how predictors were handled (10a) were reported in four and six studies respectively. Performance measures with confidence intervals (CI) (16) were only reported in 6 studies.

Mortality (7) was the most frequently predicted clinical outcome. Studies focused on mortality at different time points during follow-up, including short term (10 days [22], 30 days [28], 2 months [19], 3 months [12,27], 100 days [20]) and long term (1/3/5 years) [27]. One study [26] predicted discharge mortality (modified Rankin Score (mRS) = 6). The ML algorithms used for mortality prediction were ANN [22,28], Naïve Bayes [18,26], SVM [26,28], DT [18,19,26], and RF [26–28]. Functional outcome (measure of functional independence which relates to an individual's physical, psychological and social functioning, and the extent to which the depend on assistance from others to fulfill activities of daily living. It is usually measured by mRS) (5) was the second most commonly predicted clinical outcome. Three studies [14,15,25] predicted functional outcome (such as the ability to carry out activities of daily living e.g. washing and dressing) at 90 days, two studies [11,12] predicted it at 3 months. All of those studies used dichotomised mRS (mRS > 2 vs mRS < = 2) whilst Asadi et al. [25] also predicted 7-scale mRS (0–6). The ML algorithms used for predicting functional outcome were ANN [11,14,15,25], SVM [11,12,25], DT [12], RF [11,12,15], Super Learner [11], and Xgboost [12]. Other than mortality and mRS, Barthel Index [20,21] used RF, SVM, and kNN, hospital length of stay [18] used DT and Bayesian Network, post-stroke spasticity [25] used RF, DT, and Adaboost, neurologic deterioration [13] used SVM, quality of life [23] used ANFIS, and delayed cerebral ischemia after aneurysmal subarachnoid haemorrhage (SAH) [16] used RF were also predicted as stroke outcomes.

Among the included studies, ten studies reported having missing values, one study [28] reported no missing values, and seven studies did not mention missing values. In terms of imputation methods, complete case analysis (6) was the most commonly used among the ten studies that included information on how missing data were handled. Other imputation methods included single imputation (2) [12,24] and multiple imputation (1) [11]. For dealing with imbalanced data distributions, three studies [16,22,26] reported addressing it, of which two studies [16,26] used Synthetic Minority Over-sampling Technique (SMOTE) [16] and one study [26] did not report the method used. Four studies [12,15,16,27] did not report performing feature selection and fourteen studies reported that the features were selected before applying their algorithms.

The most commonly used ML methods were RF (9), SVM (8), DT (6), and ANN (6). The following algorithms were each used in one study: kNN [20], NB [18], BN [24], boosting [12,17], Super learner [11], and ANFIS [23]. Details of ML models is shown in S2 Text. There were fourteen models used across the studies as comparators, including logistic regression (10), Cox regression (1) [27], linear regression (1) [25], random survival forest (1) [27], and multivariate discriminant analysis (MDA) (1) [22].

For hyperparameter selection (Table 2), five studies [14,17,23,24,26] did not mention the method or rationale for hyperparameter choice, three studies [15,25,27] subjectively set a value for the hyperparameters, and ten studies performed hyperparameter tuning using the development data. Among these ten studies, grid search (5) [11,12,16,21,22] was the most widely used tuning method. Four studies [18–20,28] reported tuning hyperparameters empirically without a specific method. One study [13] used CV on the training set.

**Fig 4:**
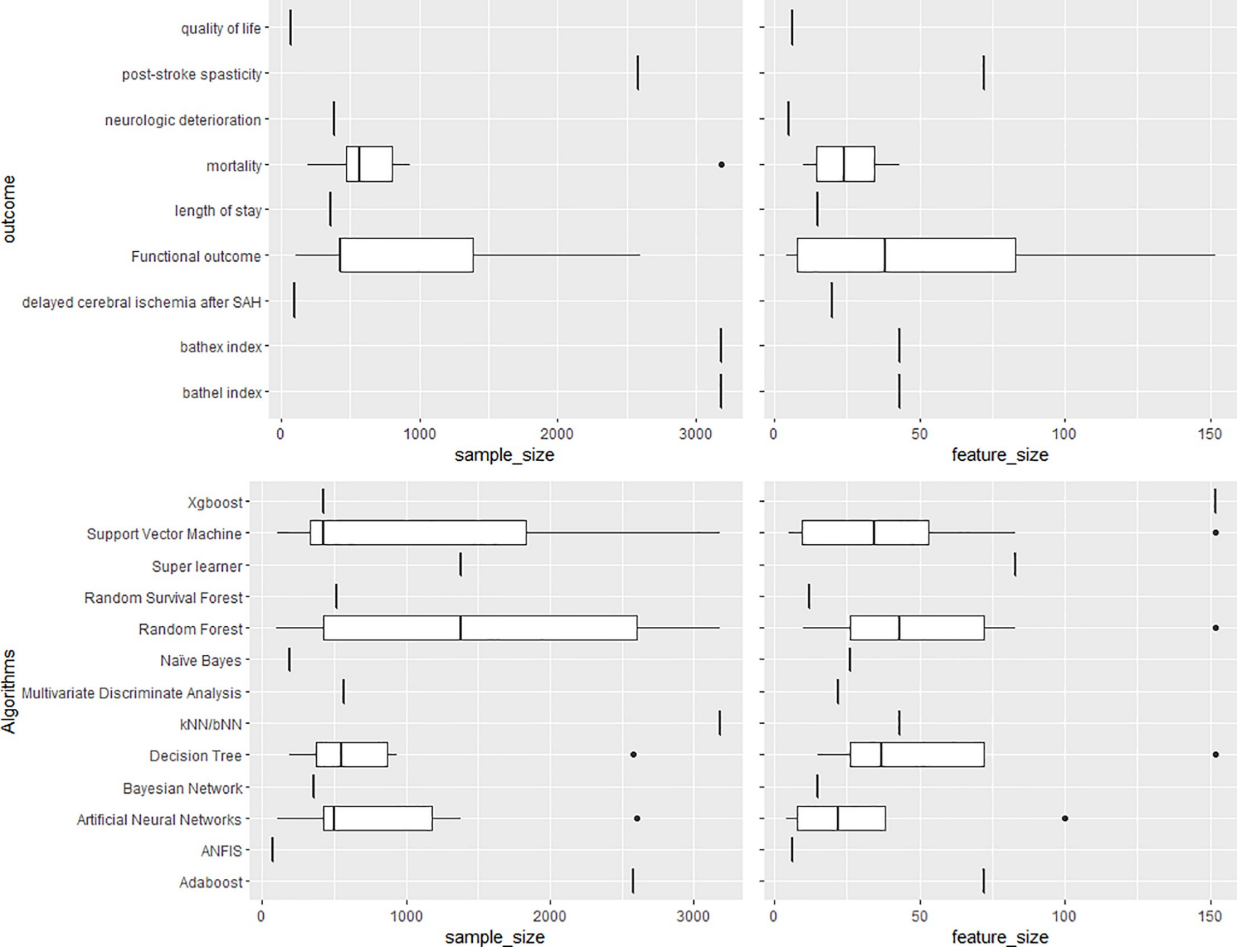
Boxplots showing the distribution of sample size and feature size according to algorithms used (top) and outcomes predicted (bottom).

There was no apparent relationship between the algorithms used and the sample size or number of features (Fig 4). Only one dataset had a sample size bigger than 3000 patients and was used by two studies [20,21]. The median sample size was 475 and the smallest was 70. The median number of features was 22 [range: 4–152].

Twelve studies compared the performance of regression models with ML algorithms (Table 2), of which six studies [12,14,15,25,26,28] reported that ML models outperformed the compared regression models and five studies [11,18,20,21,27] concluded that there was no significant difference between the ML and statistical models. One study [22] reported that LR outperformed ANN. In total, SVM outperformed the comparison algorithms in three studies [20,25,26], ANN outperformed the comparison algorithms in two studies [14,19], RF outperformed the comparisons in two studies [12,28], and LR outperformed competing algorithms in two studies [20,22].

With regards to validation methods (Table 2), CV was the most commonly used method (10) for internal validation. Eight studies split the data into training and test (and/or validation) sets. Only two studies [20,21] used external validation.

For discrimination measures, AUC (13) was the most commonly used among the classification models. Nine studies (9/13) used AUC accompanied by other discrimination measures. Four studies used only AUC. Other commonly used discrimination measures were accuracy (9), sensitivity (8), and specificity (7). Calibration was assessed in three studies [20,21,27]. One study [21] assessed calibration by plotting the observed outcome frequencies against the predicted probabilities. Two studies [20,27] used the Brier score.

## Discussion

This is the first systematic review on the application of ML methods using structured data to predict outcomes of stroke. Our results show that the interest in using ML to predict stroke outcomes using structured data has markedly increased in recent years: almost all studies in this review were published since 2014. The data sizes used in many included studies are relatively small to fully explore the potential of ML methods. Only one dataset had a sample size of over 3000 patients with a feature size of 43.

For handling missing values, almost all of the studies used only relatively simple methods such as complete case analysis and single imputation. Only one study [11] used multiple imputation. Previous studies have shown that more complicated imputation methods such as multiple imputation [29,30] are better at restoring the natural variability of the missing values than single imputation and retain more useful information than complete case analysis [31]. Future studies in the application of ML methods to stroke outcome prediction would benefit from using more sophisticated imputation methods to handle missing values.

The reporting and conducting of hyperparameter selection in the studies were often neglected though the choice of hyperparameters can greatly impact the model’s performance [32,33]. To the best of our knowledge, there exist no guidelines on reporting the hyperparameter tuning result/procedure for ML as clinical prediction models.

The most commonly used ML methods were RF, SVM, ANN, and DT. In this review, we did not compare the performance of algorithms across studies due to the different characteristics of each study. SVM performed the best in 3 studies. ANN and RF outperformed the comparison algorithms in 2 studies. Even though DTs were commonly used, they did not outperform other algorithms in the reviewed studies. The performance of ML models compared to regression models was found to be mixed, which is consistent with other ML related systematic reviews [34,35].

Performance evaluation can typically be thought to include discrimination and calibration. All studies reported discrimination whilst only three studies discussed calibration. This is concerning because poor calibration can lead to harmful decisions [36] and reporting both is essential for prediction models [37].

Validation is a crucial step for obtaining a model that can be generalised beyond the sample population. A majority of studies used internal validation methods (training and test split and CV), whilst only two studies used external validation [20,21]. External validation is an invaluable part of implementing the model in routine clinical practice–it assesses the transportability of the predictions to new data (and hence the generalisability of the model) and should be undertaken before clinical use [21,38].

None of the studies reported decision-analytic measures to assess the clinical utility of prediction models [37,39,40]. Also, no study discussed real-life implementation of the model in clinical practice even though the ultimate goal is presumably to assist the clinicians making treatment decisions and estimating prognoses. There are also several reasons why implementing ML models could be challenging in clinical settings. ML algorithms are typically not very transparent in terms of how the prediction has been made and how individual predictors have contributed to the overall prediction. This may limit the acceptability and face validity of the predictions generated by the model for clinical decision makers. In addition, we found that the reporting of the models and model building was not clear enough in most studies to enable the models to be replicated in other datasets or externally validated. This means that the models will have limited evidence of accuracy in different settings or may not be implementable at all in real-world settings.

Thus, guidelines and reporting standards for implementing ML algorithms might improve the utility of studies and future studies would benefit from attempting to evaluate potential impact and clinical utility [41]. Reporting guidelines for developing and validating clinical predictions models [7,40] provide a good starting point at this stage. Potential ethical challenges of implementing ML models was also addressed in recent studies [42]. Making algorithms and the developed models fully and publicly available with transparent and full reporting is imperative to allow independent external validation across various settings and facilitate clinical utility [43].

This systematic review has its strength and limitations. It is the first systematic review that has reviewed not only the reporting quality of the ML studies, but also the development of the ML models. Yet, even though we used published search filters for stroke, prediction models and ML, we might not have found all studies in PubMed and Web of Science, or studies that are not included in these databases and not published in English. For conference proceedings, Web of Science does include proceedings of major international conferences on machine learning such as International Conference on Machine Learning (ICML); European Conference on Machine Learning and Principles and Practices of Knowledge Discovery in Databases (ECMLPKDD); Asian Conference on Machine Learning (ACML); and International Conference on Machine Learning and Machine Intelligence (MLMI). However, there could still be smaller conferences that are not included in Web of Science.

## Conclusions

As the first systematic review on current applications of ML methods using structured data to predict outcomes of stroke, we see increasing interest in using ML for predicting stroke outcome. However, despite a surge of research articles, few met basic reporting standards for clinical prediction tools, and none of them made their models available in a way which could be used or evaluated. There is significant scope for improvement in how ML prediction algorithms are developed and validated, including using larger, richer, and more diverse data sources, improvements in model design, and fully reporting on the development process as well as the final model. As a result, it cannot be confidently said whether ML is any better than traditional statistical approaches. Major improvements in ML study conduct and reporting are needed before these methods could be meaningfully considered for practice. Guidelines and reporting standards of implementing ML algorithms could improve the utility of studies in this regard and future studies would benefit from attempting to evaluate potential impact and clinical utility.

## Supporting information

S1 Checklist The PRISMA checklist. (DOCX)

S1 Text Full PubMed and Web of Science search strategy. (DOCX)

S2 Text Summary of details of ML models used. (DOCX)

S1 Table Adjusted TRIPOD checklist for reporting quality assessment. (DOCX)

S2 Table Data extraction form. (DOCX)

S3 Table Quality assessment data for each study. (DOCX)

S4 Table Data for Publication types, venue, year, country under study, single or multi-centre study, source of data. (DOCX)

S5 Table Data for missing values reporting and handling method. (DOCX)

S6 Table Data for class imbalance level, handling method and discrimination measures. (DOCX)

S7 Table Data for feature reporting and feature selection methods. (DOCX)

S8 Table Data for publication year, data size, models used, best model, hyperparameter selection method, validation method and calibration method. (DOCX)
