# Supplementary material for: A systematic review of machine learning models for predicting outcomes of stroke with structured data
Source: PLoS One. 2020 Jun 12;15(6):e0234722. doi: 10.1371/journal.pone.0234722 (PMC7292406; doi:10.1371/journal.pone.0234722)
Supplement: S1 Text — (DOCX) [file pone.0234722.s002.docx]

**S1 Text. Search strategy**

We use PubMed and Web of Science as searching databases because PubMed remains a common and tool in biomedical electronic research, offers optimal update frequency and includes online early articles in clinical research whilst Web of Science covers science and technology which captures studies on ML applications in biomedical field. In the search strategy, we excluded publications on medical imaging as there already exist systematic reviews of the use of images for predicting outcomes of stroke with machine learning methods.3–5 We also excluded publications on stroke rehabilitation and robotics in the strategy after finding that a large amount of stroke studies were related to rehabilitation with robotics rather than prediction of clinical outcomes. We systematically excluded publications on handwriting recognition (since the word “stroke” was used in these studies to describe calligraphy).

**Pubmed search strategy:**

((Stroke[mh] OR (brain ischemia[mh:noexp] AND stroke[tiab]) OR Ischemic attack, transient[mh] OR (acute ischaemic[tiab] AND stroke[tiab]) OR (acute ischemic[tiab] AND stroke[tiab]) OR poststroke[tiab] OR post stroke[tiab] OR (cerebral hemorrhage[mh:noexp] AND stroke[tiab]) OR (carotid stenosis[mh] AND stroke[tiab])) OR (Stroke*[ti] OR 1747-4949[is] OR 1074-9357[is] OR 1524-4628[is] OR 1074-9357[is] OR 1532-8511[is] OR Brain Infarct*[tiab] OR Brain Stem Infarct*[tiab] OR Lateral Medullary Syndrome*[tiab] OR Cerebral Infarct*[tiab] OR Multi-infarct Dementia[tiab] OR Cerebral Artery infarct*[tiab] OR transient Ischemic attack*[tiab] OR transient ischaemic attack*[tiab] OR cerebral ischemi*[tiab] OR cerebrovascular event*[tiab] OR cerebrovascular accident*[tiab] OR medullary infarct*[tiab] OR cerebral artery occlusion[tiab] OR mcao[tiab] OR cerebral ischaemi*[tiab] NOT medline[sb]) AND eng[la])

AND

("Machine Learning"[Mesh] OR "Artificial Intelligence"[Mesh] OR "Natural Language Processing"[Mesh] OR "Neural Networks(Computer)"[Mesh] OR "Support Vector Machine"[Mesh] OR Machine learning[Title/Abstract] OR Artificial Intelligence[Title/Abstract] OR Naive Bayes[Title/Abstract] OR bayesian learning[Title/Abstract] OR Neural network[Title/Abstract] OR Neural networks[Title/Abstract] OR Natural language processing[Title/Abstract] OR support vector*[Title/Abstract] OR random forest*[Title/Abstract] OR boosting[Title/Abstract] OR deep learning[Title/Abstract] OR machine intelligence[Title/Abstract] OR computational intelligence[Title/Abstract] OR computer reasoning[Title/Abstract])

AND

((validate OR predict$.ti. OR rule$) OR (predict$ AND (outcome$ OR risk$ OR model$)) OR ((history OR variable$ OR criteria OR scor$ OR characteristic$ OR finding$ OR factor$) AND (predict$ OR model$ OR decision$ OR identify OR prognose)) OR (decision$ AND (model$ OR clinical$ OR logistic models/)) OR (prognostic AND (history OR variable$ OR criteria OR scor$ OR characteristic$ OR finding$ OR factor$ OR model$)) OR stratification OR ROC Curve[Mesh] OR discrimination OR discriminate OR c-statistic OR c statistic OR area under the curve OR AUC OR calibration OR indices OR algorithm OR multivariable OR (model and outcome) OR classif*)

NOT

(image[Title/Abstract] OR imaging[Title/Abstract] OR neuroimaging[Title/Abstract] OR CT[Title/Abstract] OR Computed tomography[Title/Abstract] OR MRI[Title/Abstract] OR magnetic resonance imaging[Title/Abstract] OR lesion[Title/Abstract])

NOT

(rehabilitation[Title/Abstract] OR arm[Title/Abstract] OR limb[Title/Abstract] OR hand[Title/Abstract] OR Gait[Title/Abstract] OR ankle[Title/Abstract] OR interface[Title/Abstract] OR device[Title/Abstract] OR Robo*[Title/Abstract] OR smartwatch[Title/Abstract] OR smartphone[Title/Abstract] OR phone[Title/Abstract] OR wear*[Title/Abstract] OR sensor*[Title/Abstract] OR engine[Title/Abstract] OR monitor*[Title/Abstract])

NOT

(electrocardiogram[Title/Abstract] OR ECG[Title/Abstract] OR EEG[Title/Abstract] OR signal*[Title/Abstract] OR ultrasound[Title/Abstract] OR angle*[Title/Abstract] OR gene*[Title/Abstract])

NOT

(handwrit*[Title/Abstract] OR signature[Title/Abstract])

**Web of Science search strategy:**

(TS=((cerebrovascular accident$ OR apoplexy OR brain vascular accident$ OR cva$ OR cerebral stroke$ OR Cerebral Infarction OR cerebrovascular stroke$ OR stroke$) OR ( ischemi* AND (stroke$ OR post Stroke OR poststroke)) OR ( (transient AND ischemi*) OR tia$ ) OR ( ( h$emorrhag* OR (carotid AND artery) OR (carotid AND stenos*) OR (carotid AND ulcer$)) AND (Stroke$ OR poststroke OR post stroke))))

AND

(TS=(machine learning OR Artificial Intelligence OR Naive Bayes OR bayesian learning OR Neural network$ OR Natural language processing OR support vector machine$ OR random forest$ OR boosting OR deep learning OR machine intelligence OR computational intelligence OR computer reasoning ) )

AND

(TS = ((model* and outcome$) OR classif* OR (validate OR predict* OR rule*) OR (predict* AND (outcome$ OR risk$ OR model*)) OR ((history OR variable$ OR criteria OR scor* OR characteristic$ OR finding$ OR factor$) AND (predict* OR model* OR decision$ OR identif* OR prognose$)) OR (decision$ AND (model* OR clinical$ OR logistic models)) OR (prognostic AND (history OR variable$ OR criteria OR scor* OR characteristic$ OR finding$ OR factor$ OR model*)) OR stratification OR ROC Curve OR discrimination OR discriminate OR c-statistic OR c statistic OR area under the curve OR AUC OR calibration OR indices OR algorithm$ OR multivariable) )

NOT

(TS =(imag* OR neuroimaging OR CT OR Computed tomography OR MRI OR magnetic resonance imaging OR lesion ))

NOT

(TS= (rehabilitation OR arm$ OR limb$ OR hand$ OR Gait$ OR ankle$ OR interface$ OR device$ OR Robo* OR smartwatch OR smartphone OR phone OR wear* OR sensor* OR engine OR monitor*))

NOT (TS =(electrocardiogram$ OR ECG OR EEG OR ultrasound$ OR signal* OR angle* OR gene* ))

NOT ( TS = (handwrit* OR signature))
