# Supplementary material for: A systematic review of machine learning models for predicting outcomes of stroke with structured data
Source: PLoS One. 2020 Jun 12;15(6):e0234722. doi: 10.1371/journal.pone.0234722 (PMC7292406; doi:10.1371/journal.pone.0234722)
Supplement: S2 Text — (DOCX) [file pone.0234722.s003.docx]

**S2 Text. Summary of details of ML models used**

Among the eight studies that used SVMs, five studies^23,25,29,30,32^ used a linear SVM and four studies^19,21,25,34^ used a non-linear kernel SVM. Among the six studies that used ANNs, one study^20^ used a ‘deep’ ANN with 3 hidden layers, one study^19^ used a two-layer ANN, one study^22^ used one hidden layer and three studies^18,29,30^ did not report the architecture of the ANN. Of the six studies that used DTs, three studies^17,26,33^ used the C4.5 algorithm, one study^24^ used the CART algorithm and two others^23,32^ did not mention the algorithms used. A new DT algorithm called T3 was designed^33^. Among the ten logistic regression (LR) models, five studies^18,20,22,23,26^ used standard LR, three studies^21,25,30^ explored LR with interactive terms, and two studies^29,32^ used LR with penalisation such as LASSO and Elastic Net.
