# Supplementary material for: A systematic review of machine learning models for predicting outcomes of stroke with structured data
Source: PLoS One. 2020 Jun 12;15(6):e0234722. doi: 10.1371/journal.pone.0234722 (PMC7292406; doi:10.1371/journal.pone.0234722)
Supplement: S2 Table — (DOCX) [file pone.0234722.s005.docx]

**S2 Table. Data extraction form**

| **Extracted item** | **Comments** |
| --- | --- |
| Author | Name of authors, e.g. Asadi et al. |
| Publication year | Published year of the study |
| Publication types | Journal paper, conference paper or book section |
| Published venue | Name of the journal or conference in which the study was published |
| Objective of the study | Study objectives with answering categories:  - Development and application of novel methods  - Comparison of new or existing methods  - Development of a new prediction model  - Prognostic factor study  - Validation methods study  - Other |
| Country under study population | At which country the study population was based |
| Number of centres | Answer categories:  -Single centre  -Multi-centre study  -not reported |
| Source of Data | Answer categories:  -Electronic health records  -clinical registry  -administrative data  -cohort study  -clinical trial |
| Sample size | Sample size used for building the model |
| Feature used before feature selection reported | -Yes  -No |
| Feature used for algorithms reported | -Yes  -No |
| Number of Features | Number of features for building the model |
| Were there any missing values? | Answer categories:  -not reported (did not mention)  -No (no missing values)  -Yes |
| If there were missing values, Were any variables removed from the dataset prior to the analysis because they had missing values? | Answer categories:  -n.a. (there were no missing values)  - no (no variables deleted because of missing values)  - yes if >50% of values were missing  - yes if >25% of values were missing  - yes if any value was missing |
| IF there were missing values, How were (residual) missing values handled in the analysis? | Answer categories  -n.a. (if all variables with missing values were removed in step 1; or there were no missing values in the first place)  -no, (not handled, if it is not reported, we say that it is NO, they did not handle missing values)  -complete case analysis (i.e. records with missing values were removed)  -single imputation (e.g. imputation of mean/median/most common category)  -multiple imputation (typically: MICE)  -missing values handled by the analytical method itself (e.g. decision tree; random forest) |
| Was the outcome distribution unbalanced? | Answer categories:  -Yes (if percentage of the larger class is more than 70%)  -No  -Not reported (if nothing is shown in figures, numbers, percentages) |
| If the outcome distribution was unbalanced, were steps taken to re-balance the distribution prior to the analysis? | Answer categories:  -n.a. (balanced data)  - Yes  - No (if it is not mentioned/reported to rebalance it, we assume it is No) |
| Other data preprocessing methods used | Answer categories  -No  -Yes (what has been done) |
| Clinical outcome | What is the predicted clinical outcome |
| Is statistical model used in the paper: | Answer categories:  -No  -Yes |
| Which statistical model | Answering categories:  - linear regression  -Linear regression with regularisation: Lasso, Ridge regression, Elestic net  -Logistic regression  -Logistic regression with interaction terms  -Logistic regression with regularisation  -Logistic regression with regularisation and interaction terms  -n.a. if there is no statistical model |
| Machine Leaning models | List the machine learning algorithms used |
| Were features selected prior to the actual analysis? | Answer categories:  -Yes  -No  -Not reported |
| If yes, how? | Answer categories:  -Manually, based on expert knowledge, literature review  -Data-driven methods (PCA, LASSO, Random Forest etc)  -Not reported  -n.a. |
| Hyperparameter selection | Answer categories:  -n.a. (no hyperparameters)  -Not reported  -No, no tuning was performed  -Yes, which hyperparameters were tuned and with what methods:  -Grid search/nested cross validation,  -random search,  -Bayesian optimisation etc |
| Validation methods | Answer categories  -no  Internal validation:  -Training-test splitting  - CV  -bootstraping,  external validation:  -temporal validation  -spacial validation |
| Calibration | Was calibration of risk factors examined? Answer categories:  -No  -Yes, for which algorithm and with what calibration method |
| Performance Measures | List the performance measures used |
| Did they report the importance of selected features | Answer categories:  -Yes  -No |
| Best performing model | The best performing model indicated in the study |
