# Supplementary material for: A systematic review of machine learning models for predicting outcomes of stroke with structured data
Source: PLoS One. 2020 Jun 12;15(6):e0234722. doi: 10.1371/journal.pone.0234722 (PMC7292406; doi:10.1371/journal.pone.0234722)
Supplement: S3 Table — (DOCX) [file pone.0234722.s006.docx]

**S3 Table. Quality assessment data for each study**

|  | **Methods** | | | | | | | | | | | | | | **Results** | | | | | | |
| --- | --- | --- | --- | --- | --- | --- | --- | --- | --- | --- | --- | --- | --- | --- | --- | --- | --- | --- | --- | --- | --- |
|  | **Source of Data** | | **Participants** | | **Outcome** | | **Predictors** | | **Sample Size** | **Missing Data** | **Analysis methods** | | | **Risk groups** | **Participants** | | **Model development** | | **Model specification** | | **Model performance** |
| Reference  (included studies) | 4a | 4b | 5a | 5b | 6a | 6b | Adjusted 7a | 7b | 8 | 9 | 10a | Adjusted 10b | 10d | 11 | 13a | 13b | 14a | 14b | Adjusted 15a | 15b | 16 |
| AI Taleb et al. | Yes | No | Yes | No | No | NA | No | No | No | Yes | No | No | Yes | NA | No | No | No | NA | No | No | No |
| Asadi et al. | Yes | No | No | Yes | No | No | No | No | No | No | No | No | No | NA | Yes | No | No | NA | No | No | No |
| Liang et al. | Yes | Yes | Yes | Yes | Yes | Yes | Yes | No | No | No | Yes | No | No | NA | Yes | Yes | Yes | Yes | No | No | No |
| Heo et al. | Yes | Yes | Yes | Yes | Yes | No | Yes | No | No | Yes | No | No | Yes | NA | Yes | No | Yes | NA | No | No | Yes |
| Konig et al. | Yes | Yes | Yes | No | No | No | No | No | No | Yes | Yes | Yes | Yes | NA | Yes | Yes | No | NA | No | No | Yes |
| Celik et al. | Yes | No | Yes | Yes | No | No | No | No | No | No | No | Yes | Yes | NA | Yes | No | Yes | NA | No | No | No |
| Ho et al. | Yes | Yes | Yes | Yes | No | No | No | No | No | Yes | No | No | Yes | NA | Yes | No | Yes | NA | No | No | No |
| Cox et al. | Yes | Yes | Yes | Yes | Yes | No | No | No | No | No | No | No | Yes | NA | Yes | No | No | NA | No | No | No |
| Kruppa et al. | Yes | No | Yes | No | No | No | No | No | No | Yes | No | Yes | Yes | NA | No | No | No | NA | No | No | Yes |
| Easton et al. | Yes | No | No | Yes | No | No | No | No | No | No | No | No | Yes | Yes | Yes | No | Yes | NA | No | No | No |
| Mogensen and Gerds | Yes | No | Yes | Yes | Yes | No | Yes | No | No | Yes | No | No | No | NA | Yes | No | Yes | NA | No | No | No |
| Van Os et al. | Yes | Yes | Yes | Yes | No | No | Yes | No | No | Yes | No | Yes | Yes | NA | Yes | No | Yes | NA | No | No | Yes |
| Peng et al. | Yes | Yes | Yes | Yes | No | No | Yes | No | No | NA | No | Yes | Yes | NA | Yes | Yes | Yes | NA | No | No | Yes |
| Tokmakci et al. | Yes | Yes | Yes | Yes | No | No | No | No | No | No | Yes | No | Yes | NA | Yes | Yes | No | NA | No | No | No |
| Monteiro et al. | Yes | No | Yes | Yes | No | No | No | No | No | Yes | Yes | Yes | Yes | NA | Yes | No | Yes | NA | No | No | Yes |
| Tjortjis et al. | Yes | No | No | Yes | Yes | No | Yes | No | No | Yes | Yes | Yes | No | NA | Yes | No | Yes | NA | No | No | No |
| Lin et al. | Yes | No | No | Yes | Yes | No | Yes | No | No | No | No | Yes | Yes | NA | Yes | Yes | Yes | Yes | No | No | Yes |
| Tanioka et al. | Yes | Yes | Yes | Yes | No | No | No | No | No | Yes | No | Yes | Yes | NA | Yes | Yes | No | NA | No | No | No |
| AI Taleb et al. | Yes | No | Yes | No | No | NA | No | No | No | Yes | No | No | Yes | NA | No | No | No | NA | No | No | No |
