# Supplementary material for: A systematic review of machine learning models for predicting outcomes of stroke with structured data
Source: PLoS One. 2020 Jun 12;15(6):e0234722. doi: 10.1371/journal.pone.0234722 (PMC7292406; doi:10.1371/journal.pone.0234722)
Supplement: S4 Table — (DOCX) [file pone.0234722.s007.docx]

**S4 Table. Data for Publication types, venue, year, country under study, single or multi-centre study, source of data.**

| **Reference** | **Publication types** | **Published Venue** | **Published year** | **Country under study** | **Single or multi-centre study** | **Source of data** |
| --- | --- | --- | --- | --- | --- | --- |
| Al Taleb et al. | Conference paper | International Conference on Informatics, Health & Technology (ICIHT) | 2017 | Saudi Arabia | Single centre | EHR |
| Asadi et al. | Journal paper | PLoS One | 2014 | Australia | Single centre | Clinical registry |
| Liang et al. | Journal paper | Open Med. | 2019 | China | Single centre | Cohort study |
| Heo et al. | Journal paper | Stroke | 2019 | Korea | Single centre | Cohort study |
| Konig et al. | Journal paper | Statistics in medicine | 2007 | Germany | Multi-centre | Clinical registry |
| Celik et al. | Journal paper | Journal of stroke and cerebrovascular diseases | 2014 | Turkey | Single centre | EHR |
| Ho et al. | Journal paper | AMIA Annual Symposium Proceedings | 2014 | USA | Single centre | Clinical registry |
| Cox et al. | Journal paper | Journal of Biomedical Informatics | 2016 | UK | Multi-centre | Clinical registry |
| Kruppa et al. | Journal paper | Biometrical Journal | 2014 | Germany | Multi-centre | Clinical registry |
| Easton et al. | Journal paper | Computers in Biology and Medicine | 2014 | UK | Multi-centre | Randomised control trial |
| Mogensen and Gerds | Journal paper | Statistics in Medicine | 2013 | Denmark | Multi-centre | Cohort study |
| Van Os et al. | Journal paper | Frontiers in Neurology | 2018 | Netherlands | Multi-centre | Clinical trial |
| Peng et al. | Journal paper | European Journal of Neurology | 2010 | Taiwan | Single centre | EHR |
| Tokmakci et al. | Journal paper | Expert Systems with Applications | 2008 | Turkey | Single centre | Clinical registry |
| Monteiro et al. | Journal paper | IEEE/ACM Transactions on Computational Biology and Bioinformatics | 2018 | Portugal | Single centre | Clinical registry |
| Tjortjis et al. | Journal paper | Methods of Information in Medicine | 2007 | UK | Multi-centre | Clinical registry |
| Lin et al. | Journal paper | Brain and behavior | 2018 | China | Single centre | EHR |
| Tanioka et al. | Journal paper | Molecular neurobiology | 2019 | Japan | Multi-centre | Clinical registry |
