# Supplementary material for: A systematic review of machine learning models for predicting outcomes of stroke with structured data
Source: PLoS One. 2020 Jun 12;15(6):e0234722. doi: 10.1371/journal.pone.0234722 (PMC7292406; doi:10.1371/journal.pone.0234722)
Supplement: S5 Table — (DOCX) [file pone.0234722.s008.docx]

**S5 Table. Data for missing values reporting and handling method**

| **Reference** | **missing values** | **Missingness level for imputation** | **Methods to handle missing values** |
| --- | --- | --- | --- |
| Al Taleb et al. | Yes | <50% | Cases are removed with more than 50% missing values.  Single imputation was used for handle cases with less than 50% missing values |
| Asadi et al. | Not reported | - | - |
| Liang et al. | Not reported | - | - |
| Heo et al. | Yes | 15.33% (12.86% missing outcomes) | Complete case analysis |
| Konig et al. | Yes | 1% | Complete case analysis |
| Celik et al. | Not reported | - | - |
| Ho et al. | Yes | <10% | Variables were deleted with more than 10% missing values  Then, Complete case analysis was used |
| Cox et al. | Not reported | - | - |
| Kruppa et al. | Yes | 1% | Complete case analysis |
| Easton et al. | Not reported | - | - |
| Mogensen and Gerds | Yes | Not reported | Complete case analysis |
| Van Os et al. | Yes | <25% | Variables with 25% missing values or more were discarded.  Multiple imputation by chained equations was used to impute the remaining missing values |
| Peng et al. | No | - | - |
| Tokmakci et al. | Not reported | - | - |
| Monteiro et al. | Yes | Not reported | Variables with only missing values and meta-data were deleted.  Then, single imputation was used. |
| Tjortjis et al. | Yes | Not reported | Cases were discarded with missing outcomes |
| Lin et al. | Not reported | - | - |
| Tanioka et al. | Yes | Not reported | Complete case analysis |
