# Supplementary material for: A systematic review of machine learning models for predicting outcomes of stroke with structured data
Source: PLoS One. 2020 Jun 12;15(6):e0234722. doi: 10.1371/journal.pone.0234722 (PMC7292406; doi:10.1371/journal.pone.0234722)
Supplement: S6 Table — (DOCX) [file pone.0234722.s009.docx]

**S6 Table. Data for class imbalance level, handling method and discrimination measures**

| **Reference** | **Was class distribution imbalanced?** | **Imbalance level** | **Was it handled in the study?** | **Methods to handle imbalanced class distribution** | **Performance measurements: Discrimination** |
| --- | --- | --- | --- | --- | --- |
| Al Taleb et al. | Not reported | - | - | - | Accuracy, Specificity, Sensitivity, AUC |
| Asadi et al. | Yes | <5% for mRS equals to 5 | No | - | MSE, Precision, accuracy, AUC |
| Liang et al. | No | 55% | - | - | AUC, accuracy, specificity, sensitivity |
| Heo et al. | Yes | 78% | No | - | AUC |
| Konig et al. | No | 57% | - | - | Accuracy, AUC |
| Celik et al. | Yes | 70% | Yes | Not reported | Accuracy, sensitivity, specificity |
| Ho et al. | Yes | 82% | Yes | SMOTE | c-statistic (AUC), F1-score |
| Cox et al. | Yes | 98% | No | - | Accuracy, Specificity, Sensitivity, AUC |
| Kruppa et al. | Not reported | - | - | - | AUC |
| Easton et al. | Not reported | - | - | - | AUC, specificity, sensitivity |
| Mogensen and Gerds | No | 66% | - | - | Brier score, C-index |
| Van Os et al. | No | 62% | - | - | AUC |
| Peng et al. | Yes | 85% | No | - | Accuracy, sensitivity, specificity, positive predictive value(precision), negative predictive value, +/- likelihood ratios, AUC, pairwise comparison of AUCs |
| Tokmakci et al. | -(regression) | - | - | - | Root-mean-square and mean-square error |
| Monteiro et al. | No | - | - | - | AUC |
| Tjortjis et al. | Yes | 88% | No | - | accuracy |
| Lin et al. | Yes | 73% | No | - | Sensitivity, specificity, and AUC |
| Tanioka et al. | Yes | 86% | Yes | SMOTE | accuracy, sensitivity |
