# Supplementary material for: A systematic review of machine learning models for predicting outcomes of stroke with structured data
Source: PLoS One. 2020 Jun 12;15(6):e0234722. doi: 10.1371/journal.pone.0234722 (PMC7292406; doi:10.1371/journal.pone.0234722)
Supplement: S7 Table — (DOCX) [file pone.0234722.s010.docx]

**S7 Table. Data for feature reporting and feature selection methods**

| **Reference** | **list of features before feature selection** | **List of features used for algorithm** | **Features selected before actual analysis** | **Feature selection methods** | **Feature importance reported** |
| --- | --- | --- | --- | --- | --- |
| Al Taleb et al. | No | Yes | Yes | Manually elimination redundant or irrelevant features, then backward elimination based on information gain with respect to LoS | Yes |
| Asadi et al. | No | No | Yes | Forward stepwise with Information Criterion (AICC) for entry | Yes |
| Liang et al. | Yes | Yes | Yes | Gradually variable screening with P values (unclear) | No |
| Heo et al. | Yes | Yes | No | - | No |
| Konig et al. | No | Yes | Yes for LR | Manual selection (excluded variables with a frequency of less than 4 percent in the less frequent category, and variables with substantive correlations with other variables) then use forward, backward and stepwise selection | Yes |
| Celik et al. | No | Yes | Yes | Literature review | No |
| Ho et al. | No | Yes | Yes | Manual selection (features that were available in over 90% of patient cases after the patients’ inclusion criteria), then Forward feature selection by evaluating AUC for each ML algorithm | Yes |
| Cox et al. | No | No | Yes | Manual selection (excluded treatment variables) for data trained algorithm, expert advice for clinician trained algorithm | Yes |
| Kruppa et al. | No | Yes for LR | Yes for LR | Manual selection (excluded variables with a frequency of less than 4 percent in the less frequent category, and variables with substantive correlations with other variables) then use forward, backward and stepwise selection | Yes |
| Easton et al. | Yes | No | Yes | Filter selection for Naïve Bayes,  Stepwise for LR based on Akalke information criteria | Yes |
| Mogensen and Gerds | Yes | Yes | No | - | No |
| Van Os et al. | Yes | Yes | Yes only for LR | LR: prior knowledge and expert opinions/ backward elimination/ LASSO/Elastic Net/ variable importance of RF | Yes |
| Peng et al. | Yes | Yes | Yes | No feature selection for RF,  Consistency subset evaluation (CSE) in conjunction with the exhaustive search method to select variables for ANN and SVM,  Stepwise Forward and backward selection for LR | No |
| Tokmakci et al. | No | Yes | Yes | Expert knowledge | No |
| Monteiro et al. | No | No | No | - | Yes |
| Tjortjis et al. | No | Yes | Yes | Expert knowledge | No |
| Lin et al. | No | Yes | Yes | Boruta algorithm | Yes |
| Tanioka et al. | Yes | Yes | No | - | Yes |
