# Supplementary material for: A systematic review of machine learning models for predicting outcomes of stroke with structured data
Source: PLoS One. 2020 Jun 12;15(6):e0234722. doi: 10.1371/journal.pone.0234722 (PMC7292406; doi:10.1371/journal.pone.0234722)
Supplement: S8 Table — (DOCX) [file pone.0234722.s011.docx]

**S8 Table. Data for publication year, data size, models used, best model, hyperparameter selection method, validation method and calibration method.**

| **Reference** | **Published year** | **Statistical model** | **ML Algorithms** | **Best AL** | **Sample size** | **Feature size** | **Hyperparameter selection** | **Validation method** | **Calibration method** |
| --- | --- | --- | --- | --- | --- | --- | --- | --- | --- |
| Al Taleb et. al. | 2017 | - | Decision tree: C4.5  Bayesian network | Bayesian Network | 358 | 15 | Not reported | 10-fold CV | No |
| Asadi et al. | 2014 | Linear regression | SVM with ANOVA Kernel  ANN: two-layer Feed-Forward network with sigmoid hidden and linear output neurons, | SVM | 107 | 8 | No, Manually set up | Training, test, validation for ANN,  Nested CV for SVM | No |
| Liang et al. | 2019 | Logistic regression | ANN (not reported) | ANN | 435 | 4 | Not reported | Training and test split | No |
| Heo et al. | 2019 | Logistic regression | RF (300 DT)  DNN (3 hidden layers) | DNN | 2604 | 38 | No, Manually set up | Training and test split | No |
| Konig et al. | 2007 | Logistic regression with interactive terms | RF (500 DT, 6 var per DT)  SVM with sigmoid kernel | No difference | 3184 | 43 | Yes, Experiment with different tree numbers for RF,  Grid search for SVM with tune() function in R package e1071.  No Range  No specific hyperparameters for SVM  Yes best value  method | 10-fold CV and leave one centre out CV for external validation (temporal and external validation) | Yes for LR, by plotting the observed outcome probabilities against the predicted probabiities |
| Celik et al. | 2014 | Logistic regression,  Multivariate discriminant analysis | ANN (1 hidden layer) | multivariate discriminate analysis and Log Reg | 570 | 22 | Yes, grid search (10-100 neurons)  Yes method  Range  values | 5-fold CV | No |
| Ho et al. | 2014 | Logistic regression | Naïve bayes  SVM  DT  RF  PCA+SVM | SVM | 190 | 26 | Not reported | 10-fold CV | No |
| Cox et al. | 2016 | - | RF  DT: CART  AdaBoost | RF | 2580 | 72 | Not reported | Training, test and validation split | No |
| Kruppa et al. | 2014 | Logistic regression with interactive terms | k-NN (k=87),  b-NN (bagged NN, 200 bootstrap),  SVM with linear, Bessel, Radial and Laplacian kernel,  RF (10000 DT, 3 var per DT, 15 node size per DT) | LR and SVM (linear kernel) | 3184 | 43 | Yes, For KNN, bNN and RF  KNN, bNN, RF  No range  Yes best values  Hyperparameters  No tuning for SVM | Temporal and external validation | Yes, Brier score |
| Easton et al. | 2014 | Logistic regression,  Linear regression | Naïve Bayes  DT: C4.5 | No difference | 933 | Not reported | Yes, DT is pruned | Training and test split | No |
| Mogensen and Gerds | 2013 | Cox regression, Fine-Gray model, random survival forest (1000 DT, 3 var per split) | Pseudo random forest (new, 1000 DT, 4 var per split) | No difference | 516 | 12 | No, manually set up | Bootstrap CV | Yes, Brier score |
| Van Os et al. | 2018 | Logistic regression with LASSO and Elastic Net | RF,  SVM,  ANN (not reported),  Super learner | similar between best ML and LR | 1383 | 83 | Yes, nested cross-validation with  random grid search  No range and best value | Nested CV | No |
| Peng et al. | 2010 | logistic regression with interaction terms | RF (300 DT, 5 var per DT),  ANN (not reported),  SVM | RF | 423 | 10 | Yes, (ANN chosen by software, SVM adjusted empirically)  RF not tuned | 4-fold CV | No |
| Tokmakci et al. | 2008 | - | ANFIS - Adaptive Network based Fuzzy Inference System | - | 70 | 6 | Not reported | Training and test split | No |
| Monteiro et al. | 2018 | L1 regularised logistic regression | DT,  SVM,  RF,  Xgboost | Random Forest and Xgboost | 425 | 152 | Yes, Grid search  Yes hyper parameters  Methods  No Range  Best value | 10-fold CV | No |
| Tjortjis et al. | 2007 | - | T3 (New DT proposed by authors),  DT: C4.5 | T3 | 671 | 37 | Yes, C4.5 pruned, T3 tune the stop criteria Maximum Acceptance Error (MAE) | Training and test split | No |
| Lin et al. | 2018 | - | SVM (radial basis function (RBF) kernel) | - | 382 | 5 | Yes, CV on training set with tune() function with R package e1071  No range  Yes methods, best value | Training and test split | No |
| Tanioka et al. | 2019 | - | RF (100 in model 1 and 3, 200 in model 2) | - | 95 | 20 | Yes, Grid search  Yes hyperparameters  Range, best values | Leave one out CV | No |
